# Tricho-rhino-phalangeal syndrome 1 protein functions as a scaffold required for ubiquitin-specific protease 4-directed histone deacetylase 2 de-ubiquitination and tumor growth

**DOI:** 10.1186/s13058-018-1018-7

**Published:** 2018-08-02

**Authors:** Yuzhi Wang, Jun Zhang, Lele Wu, Weiguang Liu, Guanyun Wei, Xue Gong, Yan Liu, Zhifang Ma, Fei Ma, Jean Paul Thiery, Liming Chen

**Affiliations:** 1 The Key Laboratory of Developmental Genes and Human Disease, Ministry of Education, Institute of Life Science, Southeast University, Nanjing, 210096 People’s Republic of China; 2 Jiangsu Key Laboratory for Molecular and Medical Biotechnology, College of Life Science, Nanjing Normal University, Nanjing, 210023 People’s Republic of China; 3 Cancer Science Institute, National University of Singapore, 14 Medical Drive, Singapore, Singapore; 4 Institute of Molecular and Cell Biology, A*STAR, 61 Biopolis Drive, Singapore, Singapore; 5 Department of Biochemistry, Yong Loo Lin School of Medicine, National University of Singapore, 8 Medical Drive, Singapore, Singapore

**Keywords:** TRPS1, HDAC2, USP4, De-ubiquitination, Tumor growth

## Abstract

**Background:**

Although numerous studies have reported that tricho-rhino-phalangeal syndrome type I (TRPS1) protein, the only reported atypical GATA transcription factor, is overexpressed in various carcinomas, the underlying mechanism(s) by which it contributes to cancer remain unknown.

**Methods:**

Both overexpression and knockdown of TRPS1 assays were performed to examine the effect of TRPS1 on histone deacetylase 2 (HDAC2) protein level and luminal breast cancer cell proliferation. Also, RT-qRCR, luciferase reporter assay and RNA-sequencing were used for transcription detection. Chromatin immunoprecipitation (ChIP) using H4K16ac antibody in conjunction with qPCR was used for determining H4K16ac levels in targeted genes. Furthermore, in vitro cell proliferation assay and in vivo tumor xenografts were used to detect the effect of TRPS1 on tumor growth.

**Results:**

We found that TRPS1 scaffolding recruits and enhances interaction between USP4 and HDAC2 leading to HDAC2 de-ubiquitination and H4K16 deacetylation. We detected repression of a set of cellular growth-related genes by the TRPS1-USP4-HDAC2 axis indicating it is essential in tumor growth. In vitro and in vivo experiments confirmed that silencing *TRPS1* reduced tumor growth, whereas overexpression of HDAC2 restored tumor growth.

**Conclusion:**

Our study deciphered the TRPS1-USP4-HDAC2 axis as a novel mechanism that contributes to tumor growth. Significantly, our results revealed the scaffolding function of TPRS1 in USP4-directed HDAC2 de-ubiquitination and provided new mechanistic insights into the crosstalk between TRPS1, ubiquitin, and histone modification systems leading to tumor growth.

**Electronic supplementary material:**

The online version of this article (10.1186/s13058-018-1018-7) contains supplementary material, which is available to authorized users.

## Background

TRPS1 transcription factor, the only known atypical member of GATA transcriptional factor family, contains a GATA DNA binding domain like other typical GATAs 1–6 [1]. TRPS1 is important both in development and in carcinogenesis. Mutations in TRPS1 have been documented to cause tricho-rhino-phalangeal syndrome, an autosomal-dominant disorder characterized by craniofacial and skeletal malformations [2]. Elevated TRPS1 expression has been observed in human cancers, including osteosarcoma [3], colon cancer [4], and breast cancer [5]. Recently, *TRPS1* was identified by in vivo transposon-based forward genetic screening as a potential breast cancer driver gene by our group and others [6, 7]. However, the mechanism by which TRPS1 contributes to cancer is not clear.

Histone deacetylases (HDACs) and histone acetyltranferases (HATs) are important in acetylation of histones and non-histone substrates to control and maintain a balance in the transcriptomic landscape of the normal and tumor cells [8–10]. HDACs regulate the expression and activity of numerous proteins involved in both cancer initiation and progression [10]. Eighteen mammalian HDACs have been identified and divided into four classes based on phylogenetic analysis and homology to *Saccharomyces cerevisiae* HDACs [11]. HDAC2, a member of the mammalian class I deacetylases, has been extensively studied. A decrease in HDAC2 markedly inhibits tumor growth, suggesting HDAC2 acts as an oncogene in tumorigenesis [12, 13]. Overexpression of HDAC2 protein was detected in human cancers, including gastric, prostate, and breast cancers [14, 15]. HDAC2 represses gene expression via deacetylating H4K16ac [16], determines the transcription repression program, and acts as a member of nucleosome remodeling deacetylase (NURD) complex [17].

The ubiquitin system plays a significant role in determining the fate of a protein. De-ubiquitinases (DUBs) also have fundamental roles in the ubiquitin system through deconjugating ubiquitin from the targeted proteins [18]. The ubiquitin-specific peptidase 4 (USP4) is proposed to be a potential oncogene, which can transform NIH3T3 cells [19], and USP4-deficient murine embryonic fibroblasts exhibit retarded growth [20]. Previous studies indicate that, compared to normal cells, USP4 is overexpressed in malignant cells [21]. Recently, USP4 was reported to de-ubiquitinate and stabilize HDAC2, which then inhibits p53 and NF-kB [22]. However, the mechanism by which USP4 mediates HDAC2 de-ubiquitination contributing to cancer remains unclear.

In this study, we show that the TRPS1-USP4-HDAC2 regulatory axis is involved in tumor cell proliferation. We provide a novel mechanistic insight into the growth-regulatory role of this axis by providing evidence that TRPS1 recruits USP4 to de-ubiquitinate and stabilize HDAC2. We also illustrate the scaffolding function of TRPS1 as the first example of the non-transcription factor function of GATA transcription factor which affects the ubiquitination and transcription repressive function of HDAC2, acetylation of H4K16, and the de-ubiquitinase function of USP4.

## Methods

### Cell culture

T47D, BT474, MCF7, MDA-MB-231, and HEK293T cell lines were purchased from American Type Culture Collection (ATCC) and were authenticated by the short tandem repeat (STR) typing. The cell lines were used for the current study within 6 months after cell authentication. BT474 and HEK293T cell lines were cultured in Dulbecco’s modified Eagle’s medium (DMEM) (Life Technologies, Carlsbad, CA, USA) supplemented with 10% fetal bovine serum (FBS) (HyClone, NY, USA) and 1% penicillin-streptomycin solution (Life Technologies). T47D and MCF7 were maintained in Roswell Park Memorial Institute (RPMI) 1640 medium (Corning Cellgro) supplemented with 10% FBS and 1% penicillin-streptomycin solution. To generate TRPS1 overexpression system in MDA-MB-231, the open reading frame (ORF) of TRPS1 was cloned into the lentivirus vector pCDH-CMV-MCS-EF1-copGFP (System Bioscience, CA, USA) and then transfected into HEK293T cells. The virus-containing supernatant was collected 48 h after transfection, and passed through the 0.45 μm filter to infect MDA-MB-231 cells.

### Plasmids

The following plasmids were used in this study: pCDNA3.1-Flag-USP4, pCDNA3.1-Myc-HDAC2, pCDNA3.1-His-Ub, p3 × Flag-TRPS1, p3 × Flag-TRPS1-N(1–2640), p3 × Flag-TRPS1-ΔC(1–2940), p3 × Flag-TRPS1-C(2941–3885), p3 × Flag-TRPS1-ΔN(2641–3885), p3 × Flag-TRPS1-GATA(2641–2940), 4 × UAS-TK-luciferase, GAL4-HDAC2, and Renilla luciferase (pRL-SV40).

### Antibodies

Antibodies used in this study and their sources are as follows: anti-TRPS1 (R&D Systems#AF4838), anti-HDAC2 (Cell Signaling Technology#5113), anti-H4K16ac (Millipore#39929), anti-H4 (Millipore#04–858), anti-USP4 (Cell Signaling Technology#2651), anti-actin (proteintech#60008–1-Ig), anti-HA (Biotool#B23402), anti-Flag (Sigma#F7425), anti-Myc (Biotool#B23402), anti-His (Cell Signaling Technology#12698), and anti-Gal4 (Santa Cruz#510).

### Immunoprecipitation and immunoblotting

Cells were collected and lysed for 15 min on ice in the lysis buffer (Beyotime) supplemented with a protease inhibitor. The cell lysates were incubated with antibodies and protein A/G agarose overnight at 4 °C. Unbound proteins were removed by washing three times with wash buffer. The immunoprecipitates from agarose beads were removed using the elution buffer (50 mM Tris–HCl (pH 7.4), 900 mM NaCl, 1 mM EDTA, 1% Triton X-100) for sequential co-immunoprecipitation (Co-IP). For immunoblotting following SDS-PAGE, immunoprecipitated proteins were transferred to polyvinylidene fluoride (PVDF) membranes (Millipore) and probed with various antibodies. The ECL detection system (ImageQuant LAS4000) was used for detection.

### Ubiquitination assay

To examine the ubiquitin-modified proteins, cells were lysed in the denaturing buffer (50 mM Tris-HCl pH 7.5, 150 mM NaCl, 4%SDS, 1 mM EDTA, 8% glycerol, 1 mM DTT, 1 mM PMSF and protein inhibitors) supplemented with 20 mM NEM and heated at 90 °C for 10 min. For immunoprecipitation, the lysates were further diluted to 0.1% SDS and immunoprecipitated with anti-Myc antibody at 4 °C overnight and then the ubiquitinated proteins were tested by western blotting.

### RT-qPCR analysis

For RT-qPCR analysis, total RNAs were extracted from various cell lines using the RNeasy kit (Qiagen, Hilden, Germany) and reverse transcription of RNA was performed using PrimeScript RT reagent kit (TaKaRa, Otsu, Shiga, Japan) according to the manufacturer’s instructions. The primers used for RT-qPCR are listed in Additional file 1: Table S1.

### RNA interference (RNAi)

Small interfering RNAs (siRNAs) targeting TRPS1, HDAC2, USP4 or the non-targeting control siRNA (Genepharm, Shanghai, China) were transfected into MCF7, BT474, and T47D cells using Lipofectamine RNAi MAX (Invitrogen; Carlsbad, CA, USA) according to the manufacturer’s instructions. All plasmids were transfected using Lipofectamine®2000 (Invitrogen; Carlsbad, CA, USA). An MCF7 cell line with stable depletion of TRPS1 was generated using a lentivirus short hairpin RNA (shRNA) system. The sequences for siRNAs and shRNA are listed in Additional file 2: Table S2.

### Luciferase reporter assay

HEK-293 T cells were transfected with 4 × UAS-TK-luc, Gal4-HDAC2, pRL-SV40, and Flag-TRPS1/truncations or control vector as indicated. Cells were subjected to luciferase reporter assay according to instructions provided in the Promega dual luciferase reporter assay kit.

### Cell proliferation assay

Cell proliferation was measured using the CCK-8 kit according to the protocol recommended by the manufacturer (Dojindo Laboratories, Kumamoto, Japan). Cells were seeded into 96-well plates. After treatment with siRNA, cells were grown for 24 h or 48 h. Absorbance was read at 450 nm using a Bio-Rad iMark plate reader.

### RNA-sequencing (RNA-Seq) analysis

MCF7 cells were transfected with non-targeting siRNAs (control siRNAs), TRPS1 siRNAs, and HDAC2 siRNAs. At 48 h later, total RNA was extracted from the same number of cells from each group with the RNeasy kit (OMEGA). Analysis of RNA-Seq data was performed using a standard TopHat-Cufflinks workflow.

### ChIP-qPCR

Formaldehyde was added to the cell culture medium at a final concentration of 1%, then incubated at room temperature (RT) with shaking to create protein-DNA crosslinks. After 10 min, glycine was added to the cell culture medium to stop fixation. Subsequently, the cells were washed with ice-cold PBS, harvested in SDS lysis buffer containing the protease inhibitor, and sheared by sonication. Sheared chromatin was used for immunoprecipitation with IgG and anti-H4K14Ac antibodies. The immunoprecipitates were washed, reverse crosslinked, and eluted to obtain the purified DNA for q-PCR. Primer sequences used for ChIP-qPCR experiments are listed in Additional file 1: Table S1.

### Tumor xenografts

Four-to-six-week-old female athymic nude (Foxn/nu/nu) mice were purchased from the Model Animal Research Center of Nanjing University. MCF-7 cells (5 × 10^6^) suspended in 200 μl of the PBS–Matrigel mixture were injected into the mammary fat pads. A 0.72-mg E2 60-day release pellet (Innovative Research of America, Sarasota, FL, USA) was implanted subcutaneously on the dorsal side of each mouse a day before tumor cells injection. The length and width of tumors were examined weekly using a Vernier caliper and the volume was calculated by the formula:

π/6 × length×width^2^.

## Results

### TRPS1 regulates HDAC2 protein level by stabilizing HDAC2 in ubiquitin-dependent proteasomal degradation (UDPD)

**Fig. 1:**
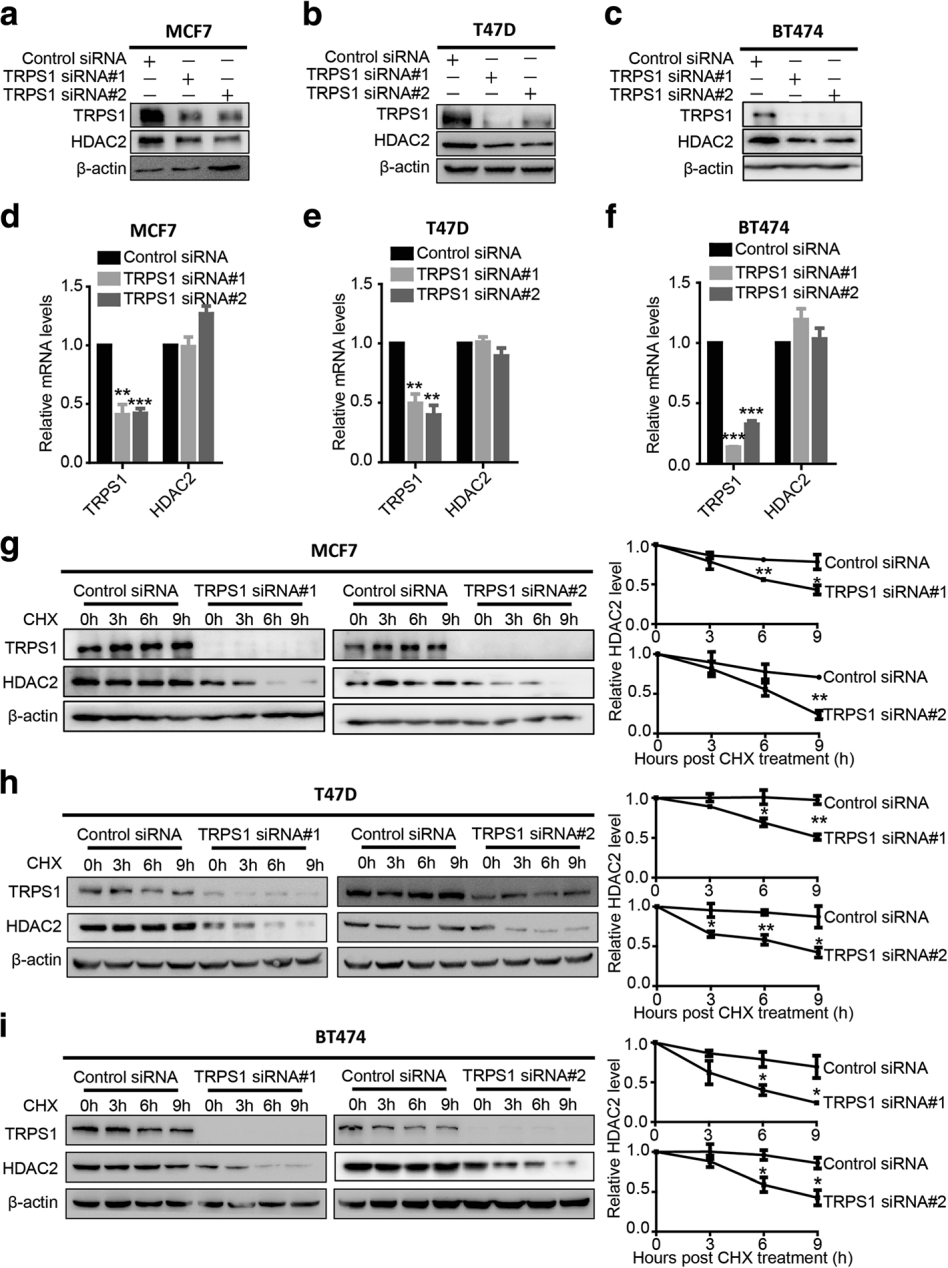
Tricho-rhino-phalangeal syndrome 1 (TRPS1) transcription factor negatively regulates histone deacetylase 2 (HDAC2) protein level by stabilizing the HDAC2 protein. MCF7 (**a**), T47D (**b**), and BT474 (**c**) exhibit decreased HDAC2 protein levels upon silencing *TRPS1*. MCF7 (**d**), T47D (**e**), and BT474 (**f**) exhibit insignificant alterations in *HDAC2* messenger RNA levels upon silencing *TRPS1*. MCF7 (**g**), T47D (**h**), and BT474 (**i**) show decreased HDAC2 protein stability upon silencing *TRPS1*. The *t* test was used for statistical quantification: *p < 0.05, **p < 0.01, ***p < 0.001, respectively. siRNA, small interfering RNA; CHX, cycloheximide

Analysis of TRPS1 interactome had indicated that TRPS1 is associated with HDAC1 and HDAC2 [23], which belong to class I HDACs. To explore the function of TRPS1, we used luminal breast cancer cells MCF7, T47D, and BT474 with elevated TRPS1 as model cell lines. Silencing *TRPS1* with two different siRNAs in all three cell lines consistently showed decreased HDAC2 but not HDAC1 protein levels indicating TRPS1 positively regulates HDAC2 and not HDAC1 (Fig. 1a–c and Additional file 3: Figure S1A–C). To further confirm this observation, we overexpressed *TRPS1* in MDA-MB-231 cells with no detectable endogenous TRPS1 expression and detected increased HDAC2 protein levels with ectopic overexpression of *TRPS1* (Additional file 3: Figure S1D). Since TRPS1, a member of the GATA transcription factor family, is a well-documented transcription factor, we first investigated whether TRPS1 transcriptionally regulated the expression of *HDAC2*. We found that neither knockdown nor overexpression of *TRPS1* was able to significantly change *HDAC2* mRNA levels (Fig. 1d–f and Additional file 3: Figure S1E). These observations suggested that TRPS1 regulated HDAC2 protein level independent of its transcription factor function. We then hypothesized that TRPS1 regulates HDAC2 protein level by affecting its protein stability. To test this idea, we first treated cells with cycloheximide (CHX), a eukaryotic protein synthesis inhibitor, and found that in the presence of CHX, silencing of *TRPS1* significantly reduced HDAC2 protein stability (Fig. 1g–i). Conversely, overexpression of *TRPS1* in cells increased HDAC2 stability (Additional file 3: Figure S1F). These results suggested that TRPS1 positively regulated and stabilized HDAC2 protein level.

**Fig. 2:**
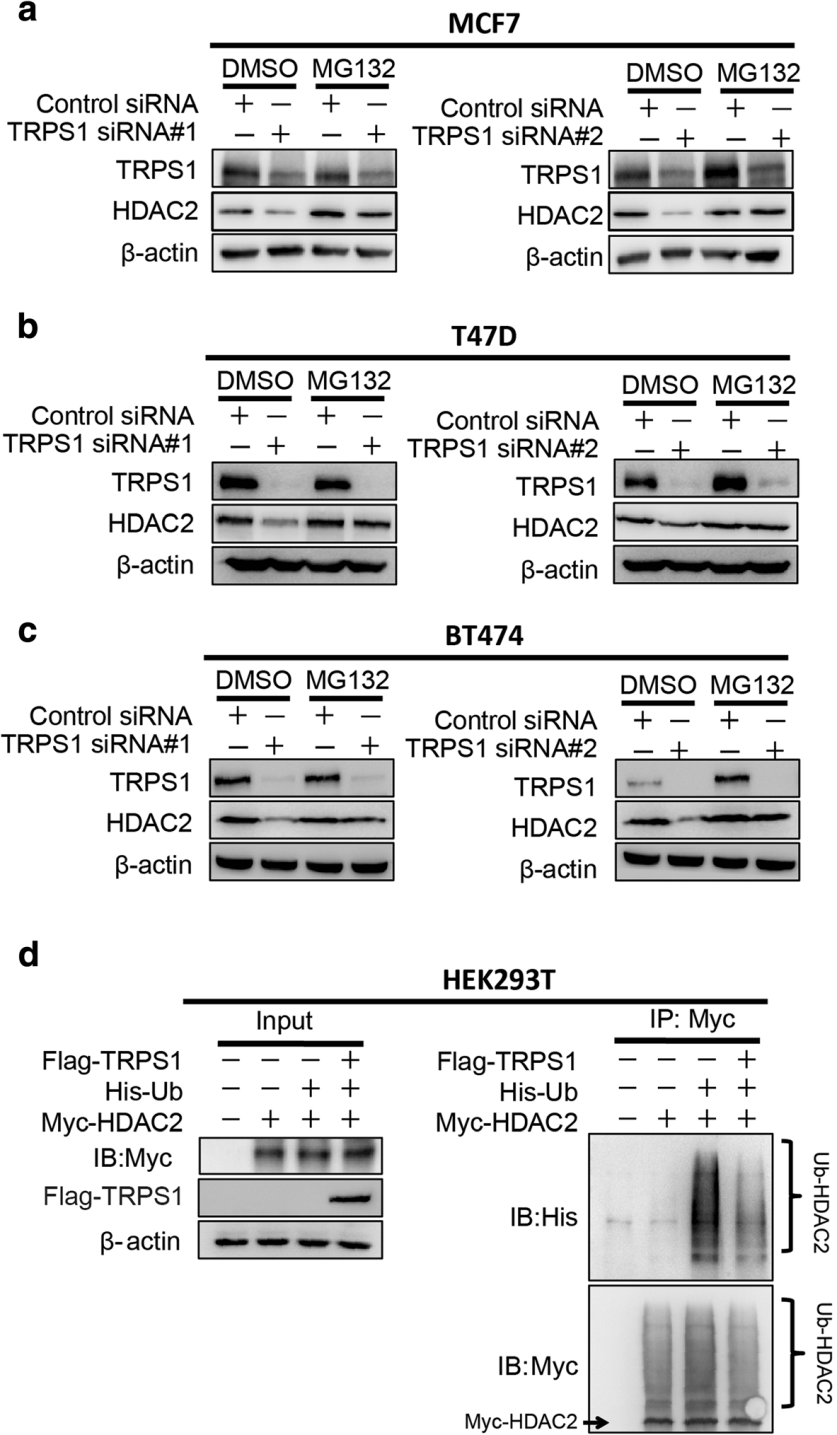
Tricho-rhino-phalangeal syndrome 1 (TRPS1) transcription factor stabilizes histone deacetylase 2 (HDAC2) through ubiquitin-dependent proteasomal degradation (UDPD). MCF7 (**a**), T47D (**b**), and BT474 (**c**) show decreased HDAC2 protein levels upon MG132 treatment in cells with silencing of *TRPS1*. **d** Ectopic overexpression of TRPS1 reduced HDAC2 ubiquitination level in HEK293T. DMSO, dimethyl sulfoxide; siRNA, small interfering RNA

It is well-known that the majority of intracellular proteins are degraded by UDPD [24]. To test whether TRPS1 stabilized HDAC2 levels by modulating UDPD, we used MG132, a specific 26-s proteasome inhibitor with the ability to reduce the degradation of ubiquitin-conjugated proteins in mammalian cells. Fig. 2a–c shows that HDAC2 protein levels were sustainable in the presence of MG132 if we silenced *TRPS1*. To further confirm if TRPS1 inhibited HDAC2 in a UDPD-dependent manner, we performed ubiquitination assay by co-transfecting HEK293T cells with Myc-HDAC2 vector plus His and Flag-empty vectors, or His-Ubiquitin and Flag empty vectors, or His-Ubiquitin and Flag-TRPS1 vector and compared these with Myc-empty vector plus His and Flag empty vectors. At 24 h later, we immunoprecipitated HDAC2 from whole cell lysates using an anti-Myc antibody, and evaluated HDAC2 ubiquitination (Ub-HDAC2) using anti-His antibody. We found that TRPS1 overexpression reduced HDAC2 ubiquitination level (Fig. 2d). Taken together, these results suggested that TRPS1 stabilized HDAC2 protein levels by reducing the UDPD of HDAC2.

### TRPS1 functions as a scaffold protein to recruit UPS4 and HDAC2 to de-ubiquitinate and stabilize HDAC2

**Fig. 3:**
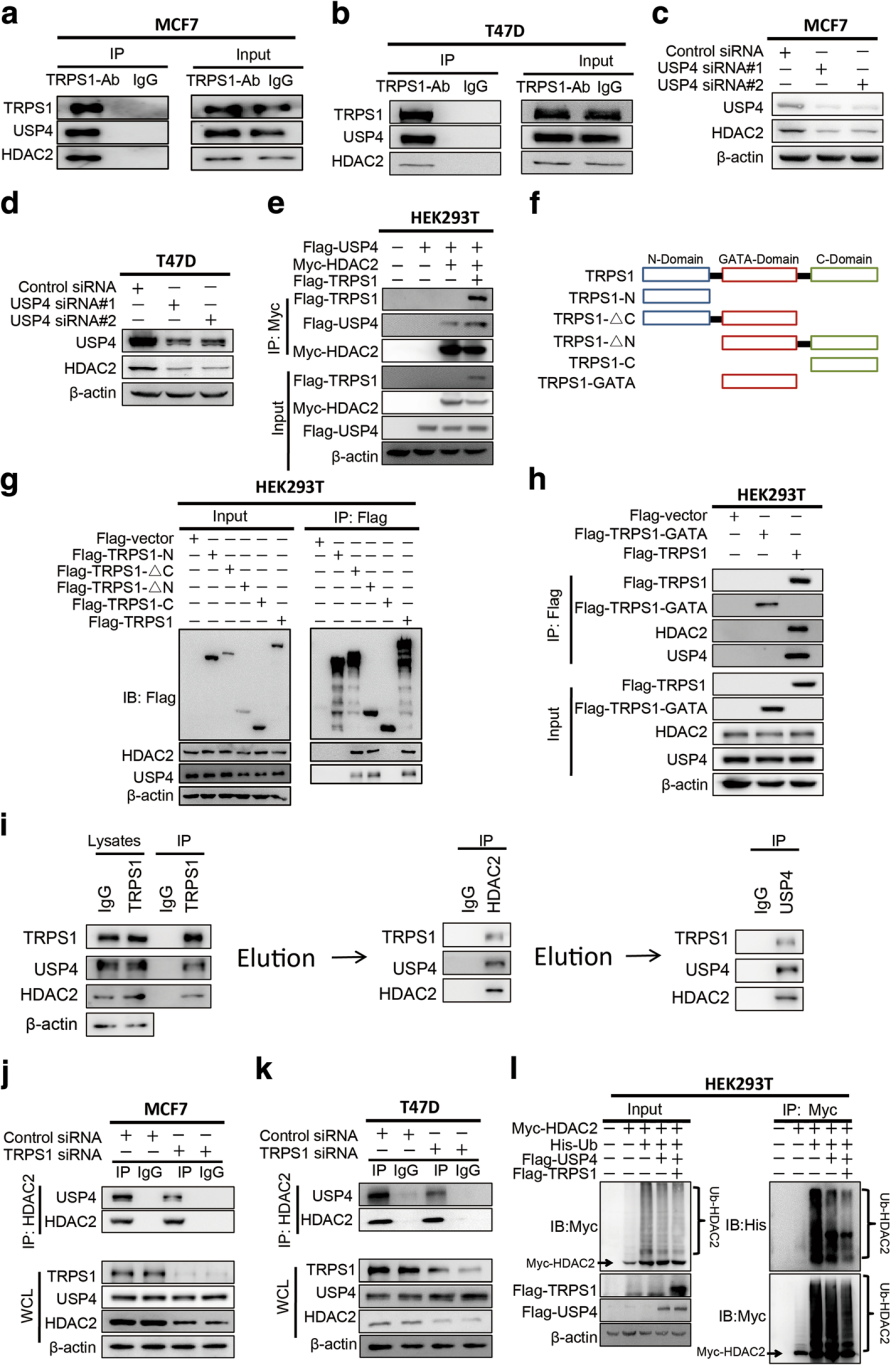
Tricho-rhino-phalangeal syndrome 1 (TRPS1) transcription factor recruits ubiquitin-specific protease 4 (USP4) and histone deacetylase 2 (HDAC2) to form a complex in which HDAC2 is de-ubiquitinated by USP4. MCF7 (**a**) and T47D (**b**) show co-immunoprecipitation (Co-IP) of TRPS1, USP4 and HDAC2. MCF7 (**c**) and T47D (**d**) exhibit decreased HDAC2 protein levels upon silencing of *USP4*. **e** Co-IP analysis of the interaction between TRPS1, USP4, and HDAC2 in HEK293T cells with ectopic overexpression of Flag-TRPS1, Flag-USP4, and Myc-HDAC2 using anti-Myc antibody. **f** Domain structures of TRPS1 and its truncation mutants. **g** Co-IP analysis of the interaction between truncation mutants of TRPS1 and USP4 and HDAC2 in HEK293T cells with ectopic overexpression of TRPS1 and its truncation mutants. **h** Co-IP analysis of the interaction between the GATA domain of TRPS1 and USP4 and HDAC2 in HEK293T cells with ectopic overexpression of TRPS1 GATA domain truncation mutant. **i** MCF7 cell lysates were subjected to immunoprecipitation with control IgG or anti-TRPS1 antibody. The immunoprecipitates bound to agarose beads were eluted by elution buffer and sequential immunoprecipitation was performed with the indicated antibodies. **j** MCF7 and **k** T47D show reduced interaction between USP4 and HDAC2 upon silencing TRPS1 in Co-IP assay using anti-HDAC2 antibody. **l** Overexpression of USP4 decreased HDAC2 ubiquitination level and additional overexpression of TRPS1 enhanced reduction of HDAC2 ubiquitination level in HEK293T cells

To test whether TRPS1 inhibits UDPD of HDAC2 through directly binding to HDAC2, we performed Co-IP using anti-TRPS1 antibody. As displayed in Fig. 3a, b, the results confirmed that TRPS1 physically associated with HDAC2. Since TRPS1 is not a ubiquitin-specific peptidase that could directly de-ubiquitinate and stabilize its interacting partner HDAC2, we hypothesized that TRPS1 reduced HDAC2 ubiquitin level by a ubiquitin-specific peptidase to de-ubiquitinate HDAC2 by reducing UDPD of HDAC2. It has recently been reported that USP4 directly interacts and de-ubiquitinates HDAC2 resulting in its stability in colon cancer cells [22]. To test whether USP4 was responsible for TRPS1-mediated HDAC2 de-ubiquitination and stabilization, we first tested whether, like TRSP1, USP4 had a stabilizing effect on HDAC2. By silencing *USP4* in T47D and MCF7 cells, we detected decreased HDAC2 protein levels as was the case with TRPS1 (Fig. 3c, d).

Since TRPS1 is well-documented as a transcription factor, we examined whether *USP4* is transcriptionally regulated by TRPS1. We investigated USP4 expression upon silencing TRPS1 and found no change in either USP4 mRNA or protein levels (Additional file 4: Figure S2A, B). It appeared that both TRPS1 and USP4 interacted with and stabilized HDAC2 in a UDPD-dependent fashion. We hypothesized that TRPS1, USP4, and HDAC2 formed a complex, used Co-IP to test this notion, and found that TRPS1 co-immunoprecipitated with USP4 and HDAC2 (Fig. 3a, b). To further validate this point, we co-transfected Flag-USP4, Myc-HDAC2, and Flag-TRPS1 in HEK293T cells and performed Co-IP using anti-Myc antibody and found that TRPS1, USP4, and HDAC2 were co-purified as a complex (Fig. 3e).

To further confirm the interaction between TRPS1, USP4, and HDAC2, we generated serial TRPS1 truncates based on the domain structure of TRPS1 consisting of an N-terminal domain and GATA and C-terminal domains (Fig. 3f). We performed Co-IP experiments in HEK293T cells with ectopic overexpression of TRPS1 or TRPS1 truncation mutants. The results indicated that N-terminal or C-terminal domains of TRPS1 together with the GATA domain were sufficient to interact with HDAC2 and USP4 while neither TRPS1 N-terminal or C-terminal domain alone interacted with HDAC2 and USP4 (Fig. 3g). These observations indicated that GATA-zinc finger domain of TRPS1 was essential for its interactions with HDAC2 and USP4. To test whether the TRPS1 GATA domain alone was sufficient to interact with USP4 or HDAC2 or both, we carried out Co-IP experiments in HEK293T cells with ectopic overexpression of TRPS1-GATA truncate containing only the GATA-zinc finger domain. Our results indicated that the GATA domain of TRPS1 alone was unable to interact with HDAC2 and USP4 (Fig. 3h). These observations suggested that the GATA-zinc finger domain of TRPS1 was necessary but not sufficient for TRPS1, USP4, and HDAC2 complex formation. Interestingly, although ectopically overexpressed Flag-UPS4 could be co-immunoprecipitated with overexpressed Myc-HDAC2 using anti-Myc antibodies, overexpression of TRPS1 could increase the co-immunoprecipitated UPS4 levels in this experiment (Fig. 3e). These findings raised the possibility that TRPS1 functions as a scaffold protein to facilitate the UPS4 and HDAC2 interaction. To test this notion, we first performed a sequential immunoprecipitation assay and confirmed that TRPS1, USP4, and HDAC2 formed a ternary complex (Fig. 3i). Furthermore, when we silenced *TRPS1* in cells and carried out Co-IP using anti-HDAC2 antibodies, the co-immunoprecipitated USP4 level with HDAC2 decreased significantly (Fig. 3j, k). We further verified that TRPS1 functioned as a scaffold protein for the complex formation of TRPS1-USP4-HDAC2 and was responsible for the reduction of HDAC2 ubiquitination level by facilitating interaction between USP4 and HDAC2. We carried out ubiquitination assays by co-transfecting HEK293T cells with a Myc-HDAC2 expression plasmid plus His and Flag empty vectors, His-Ubiquitin and Flag empty vectors, His-Ubiquitin, Flag-USP4 and Flag empty vectors,or His-Ubiquitin, Flag-USP4 and Flag-TRPS1 vectors and compared them with cells transfected with Myc plus His vector and Flag-empty vectors. Although USP4 alone could reduce HDAC2 ubiquitination level, additional overexpression of TRPS1 significantly enhanced the reduction of HDAC2 ubiquitination (Fig. 3l). Taken together, our results suggested that TRPS1 stabilized HDAC2 by functioning as a scaffold protein to bring UPS4 and HDAC2 together forming the TRPS1-UPS4-HDAC2 complex to enhance USP4-directed HDAC2 de-ubiquitination.

### TRPS1-UPS4-HDAC2 axis regulates transcriptional repression activity of HDAC2

**Fig. 4:**
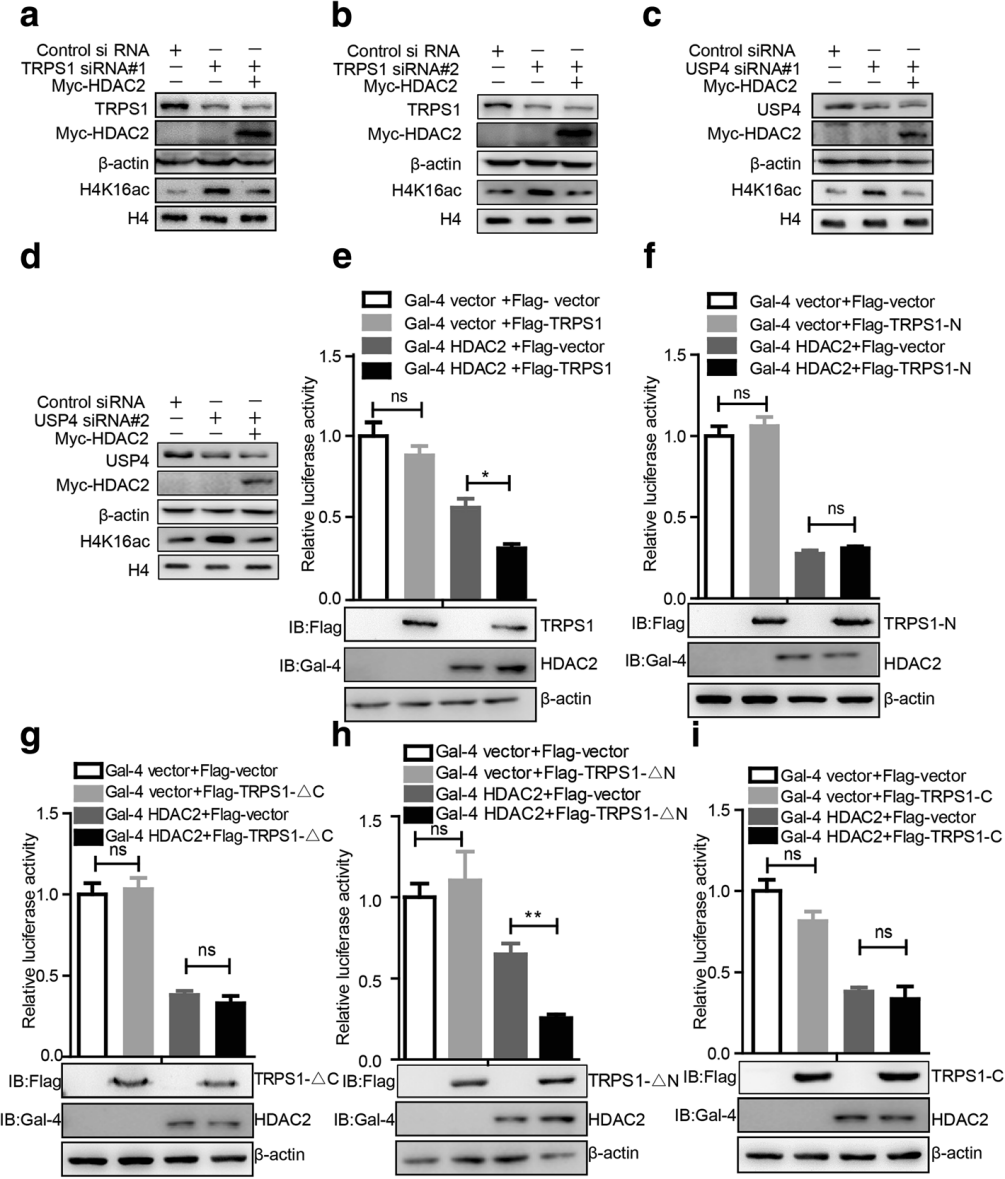
Tricho-rhino-phalangeal syndrome 1 (TRPS1) transcription factor regulates the transcription repressive activity of histone deacetylase 2 (HDAC2). **a**, **b** MCF7 shows increased H4K16ac level upon silencing of *TRPS1,* and overexpression of HDAC2 restored H4K16ac level. c, d MCF7 shows increased H4K16ac level upon silencing *USP4,* and overexpression of HDAC2 restored H4K16ac level. **e**–**i** Effects of TRPS1 and its truncation mutants over the transcriptional repression activity of HDAC2. The *t* test was used for statistical analysis: *p < 0.05; ns, not significant. Ab, antibody; siRNA, small interfering RNA; USP4, ubiquitin-specific protease 4

HDAC2, as a histone deacetylase, exerts its transcriptional repression activity by deacetylating histones [25]. H4K16ac, which is a key epigenetic marker and is necessary for transcriptional regulation [26], has been shown to be a major deacetylation target of HDAC2 [16]. Consistent with these notions, we observed that silencing of *HDAC2* in T47D and MCF7 cells led to increased H4K16ac levels (Additional file 5: Figure S3A, B). Furthermore, silencing *TRPS1* increased H4K16ac level as did silencing *HDAC2* (Additional file 5: Figure S3C–E). These observations indicated that TRPS1-UPS4-HDAC2 formed a functional axis in controlling the acetylation status of H4K16 via HDAC2. To further confirm this notion, we first tested whether TRPS1 controlled the acetylation status of H4K16 via HDAC2. We silenced *TRPS1* with siRNAs with or without rescuing overexpression of HDAC2 in MCF7 and found that silencing *TRPS1* led to decreased HDAC2 and increased H4K16ac, whereas HDAC2 overexpression restored the H4K16ac level (Fig. 4a, b). Furthermore, when we silenced *USP4* with siRNAs with or without HDAC2 overexpression in MCF7, we consistently found that USP4 silencing led to decreased HDAC2 and increased H4K16ac, whereas additional HDAC2 overexpression restored the H4K16ac level (Fig. 4c, d). Reduced H4K16ac has been shown to be an indicator of transcription repression [27–29]. Gal4-TK-luciferase reporter assay is generally used to test HDAC2 transcriptional repression activity [30–33]. To further investigate whether TRPS1 could affect HDAC2 transcriptional repression activity, we co-transfected the Gal4-TK-luciferase plasmid with Gal4-HDAC2 with or without Flag-TRPS1 and TRPS1 domain truncates. As shown in Fig. 4e–i, ectopic overexpression of full-length TRPS1 and TRPS1-△N truncate exhibited strong repression activity of luciferase expression while TRPS1-N, TRPS1-△C, and TRPS1-C truncates did not show significant effects. These observations indicated that TRPS1 regulated transcriptional repression activity of HDAC2. Furthermore, the C-terminal domain of TRPS1 was necessary but not sufficient for transcriptional repression activity of HDAC2. This could be due to the fact that C-terminal and GATA domains of TRPS1 were required for binding of TRPS1 to HDAC2 (Fig. 3). As we proposed, TRPS1 recruited UPS4 to mediate HDAC2 stability, and regulated its transcriptional repression activity (Additional file 5: Figure S3F).

**Fig. 5:**
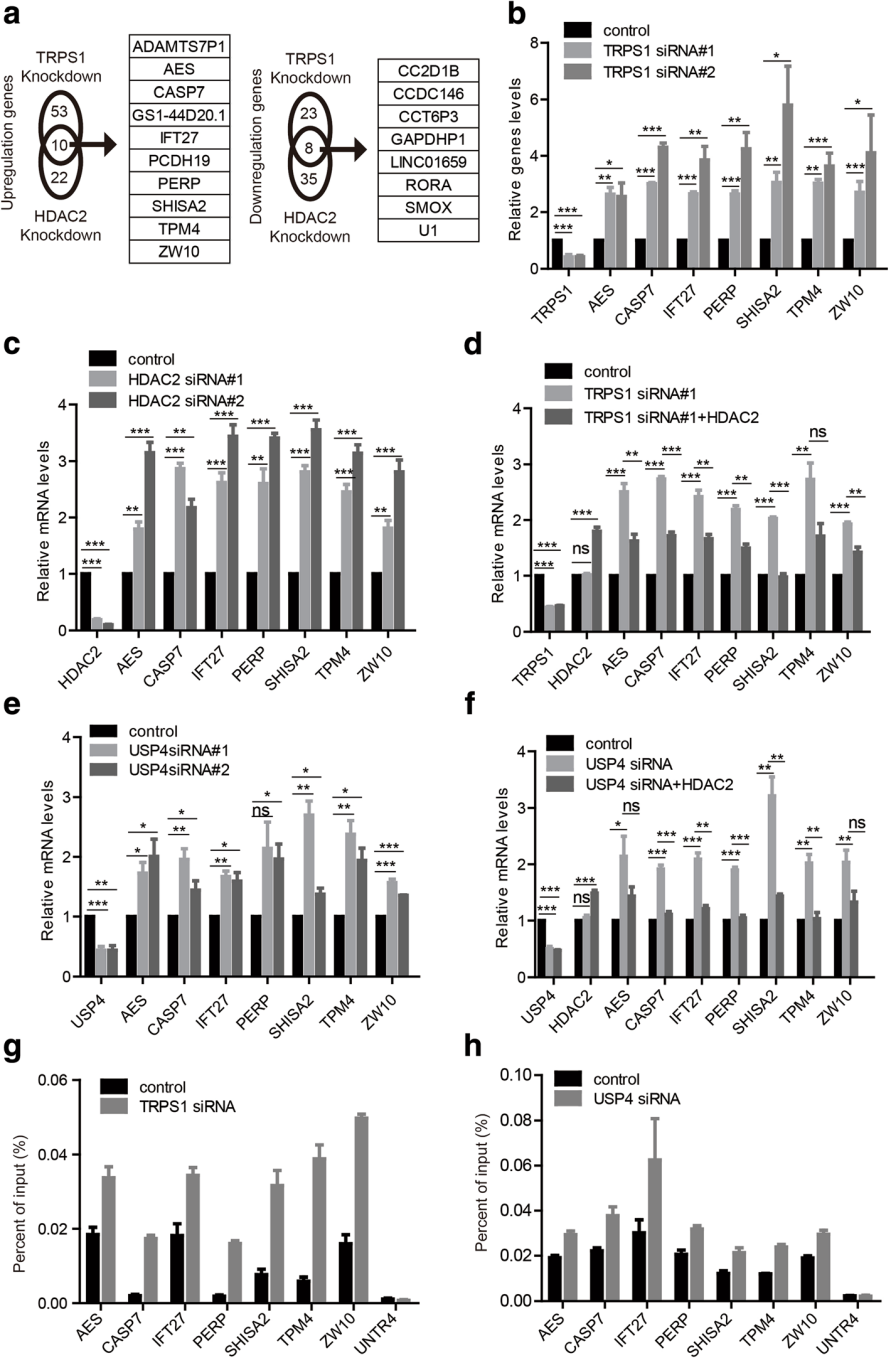
Transcription output analysis of the tricho-rhino-phalangeal syndrome 1 (TRPSI)-ubiquitin-specific protease 4 (USP4)-histone deacetylase 2 (HDAC2) regulatory axis. **a** Analysis and comparison of transcriptomes of MCF7 upon silencing *TRPS1* or *HDAC2* using RNA sequencing. **b** Validation of the selected TRPS1 target genes using RT-qPCR. **c** Validation the selected HDAC2 target genes using RT-qPCR. **d** Expression levels of genes up-regulated upon silencing of *TRPS1* were restored with overexpression of HDAC2. **e** Selected genes upon silencing of *TRPS1* or *HDAC2* show consistent up-regulation by RT-qPCR upon silencing of *USP4* in MCF7. **f** Expression levels of genes up-regulated upon silencing of *USP4* were restored with additional overexpression of HDAC2. **g**, **h** Chromatin immunoprecipitation (ChIP)-qPCR using H4K16AC antibodies on selected target genes upon silencing of *TRPS1* or *USP4* in MCF7 cells. The *t* test was used for statistical quantifications: *p < 0.05, **p < 0.01, ***p < 0.001, respectively. SiRNA, small interfering RNA

To further identify the transcriptional output of HDAC2 mediated by TRPS1, we first investigated transcriptional alterations by RNA sequencing in MCF7 cells. Upon silencing of *TRPS1* in MCF7 cells, 66 and 33 genes were up- and down-regulated, respectively (fold change ≥ 2 and *q* < 0.05), and upon silencing *HDAC2* in MCF7 cells, 23 and 44 genes were up-regulated and down-regulated, respectively (fold change ≥ 2 and *q* < 0.05) (Additional file 6: Table S3A, B). There were 10 genes, *ADAMTS7P1*, *AES*, *CASP7*, *GS1-44D20.1*, *IFT27*, *PCDH19*, *PERP*, *SHISA2*, *TPM4,* and *ZW10*, that were consistently upregulated upon silencing either *TRPS1* or *HDAC2*, while 8 genes, *CC2D1B*, *CCDC146*, *CCT6P3*, *GAPDHP1*, *LINC01659*, *RORA*, *SMOX,* and *U1* were consistently down-regulated upon silencing either *TRPS1* or *HDAC2* (Fig. 5a). The genes with consistently up-regulated expression upon silencing either *TRPS1* or *HDAC2* were considered as candidate targets for *TRPS1* transcriptional repressive function over *HDAC2*. RT-qPCR validated that *AES*, *CASP7*, *IFT27*, *PERP*, *SHISA2*, *TPM4*, and *ZW10* were consistently up-regulated upon silencing either *TRPS1* or *HDAC2* (Fig. 5b, c). Furthermore, additional overexpression *HDAC2* in cells with *TRPS1* silencing restored the expression levels of these genes (Fig. 5d). Considering the function of the regulatory axis of TRPS1, USP4, and HDAC2 on transcriptional regulation, we further confirmed that silencing USP4 led to consistent up-regulation of *AES*, *CASP7*, *IFT27*, *PERP*, *SHISA2*, *TPM4*, and *ZW10,* and additional overexpression of HDAC2 restored the expression levels of these genes (Fig. 5e, f). To determine the H4K16ac level at the affected genes, we carried out ChIP-qPCR using H4K16ac antibody upon silencing *TRPS1* or *USP4* and found that silencing either TRPS1 or USP4 alone increased the H4K16ac level at these target genes (Fig. 5g, h). Taken together, these results support our claim that the TRPS1-UPS4-HDAC2 axis regulates transcriptional repression activity of HDAC2.

### TRPS1-UPS4-HDAC2 axis confers tumor growth

**Fig. 6:**
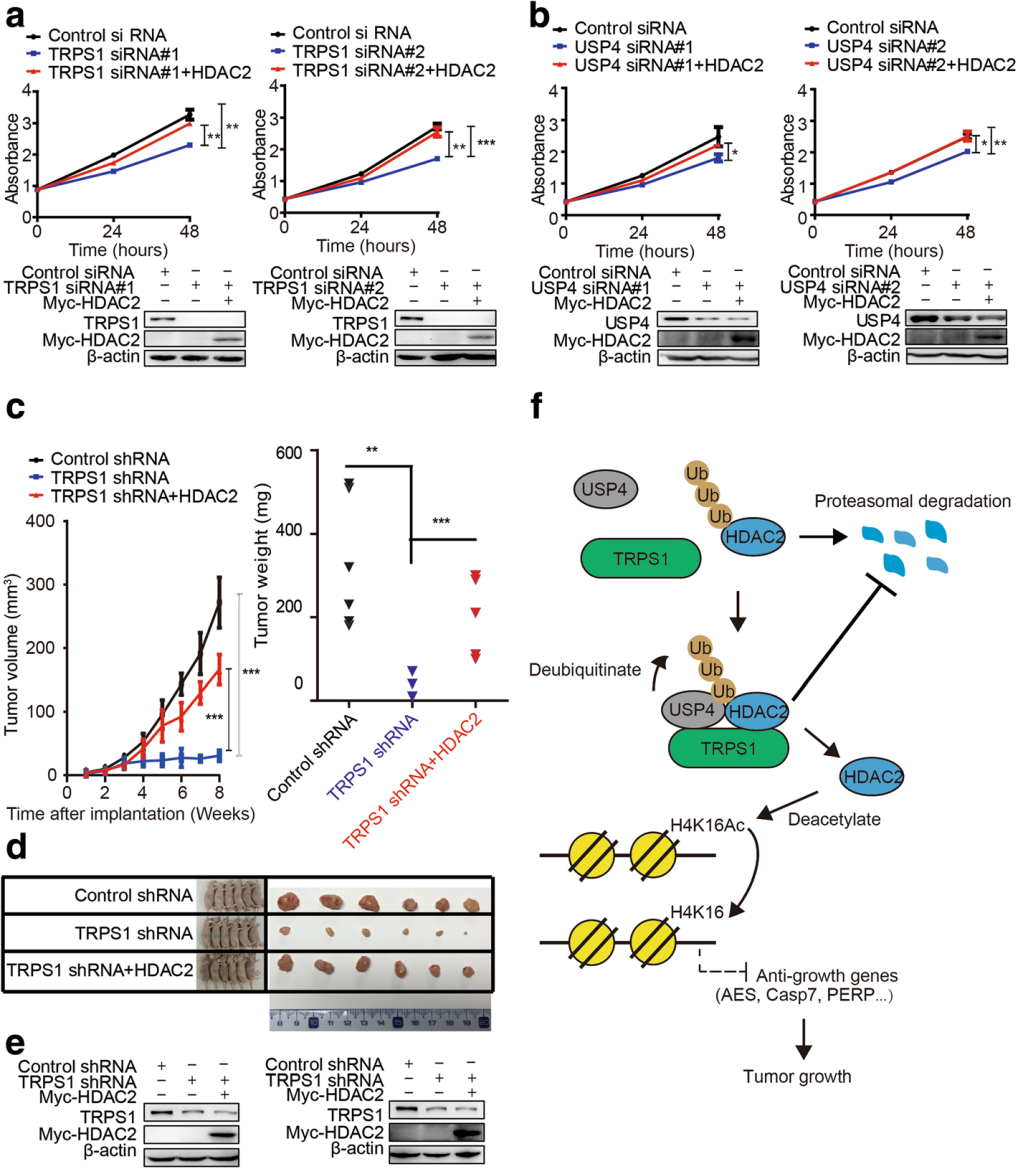
The tricho-rhino-phalangeal syndrome 1 (TRPSI)-ubiquitin-specific protease 4 (USP4)-histone deacetylase 2 (HDAC2) regulatory axis confers growth in cancer cells in vitro and in xenografted tumors in vivo. **a** MCF7 shows decreased cell viability upon silencing of *TRPS1* and additional overexpression of HDAC2 restored cell viability. **b** MCF7 shows decreased cell viability upon silencing of *USP4* and additional overexpression of HDAC2 restored cell viability. **c** Xenografted tumor growth curves (left), xenografted tumor weight (right). **d** Representative xenografted tumors from mouse models. **e** Representative western blot data of selected xenografted tumors show TRPS1 and myc-HDAC2 protein levels. **f** A working model of the current study. The *t* test was used for statistic quantifications: *p < 0.05, **p < 0.01, ***p < 0.001, respectively. siRNA, small interfering RNA

The set of genes consisting of *AES*, *CASP7*, *IFT27*, *PERP*, *SHISA2*, *TPM4*, and *ZW10,* repressed by the TRPS1-USP4-HDAC2 axis were implicated in cell proliferation [34–40]. Thus, we postulated that the TRPS1-USP4-HDAC2 axis contributes to tumor growth. To test this idea, we first performed in vitro cell culture experiments. Silencing of *TRPS1* reduced proliferation of MCF7 cells while overexpression of HDAC2 in cells with TRPS1 silencing rescued this phenotype (Fig. 6a). Similarly, silencing of *USP4* also reduced proliferation of MCF7 while overexpression of HDAC2 in cells with *USP4* silencing rescued this phenotype (Fig. 6b).

Consistent with the in vitro experiments, in xenograft mouse models, silencing *TRPS1* significantly reduced both tumor volume and weight, and additional overexpression of HDAC2 rescued this phenotype of xenograft tumors (Fig. 6c–e). Thus, both in vitro and in vivo results demonstrated that the TPRS1-USP4-HDAC2 regulatory axis confers tumor growth.

Taken together these results led to a mechanistic scheme in which TRPS1 scaffolding recruits USP4 to de-ubiquitinate and stabilize HDAC2, leading to repression of a set of anti-growth genes and acceleration of tumor growth (Fig. 6f).

## Discussion

In this study, we report that TRPS1, USP4, and HDAC2 form a regulatory axis to confer tumor growth. TRPS1 acts as a scaffold protein in this axis and recruits USP4 and HDAC2 leading to de-ubiquitination and stabilization of HDAC2, deacetylation of H4K16, and transcriptional repression of anti-proliferative genes.

TRPS1, the only reported atypical GATA transcription factor, had been characterized as the first example of a GATA protein with intrinsic transcriptional repression activity [1]. Our results provide insight into the non-transcription factor function of GATA transcription factors by discovering the scaffolding effect of TRPS1 in USP4-mediated HDAC2 de-ubiquitination and, for the first time, furnish evidence of the physical association and functional link between TRPS1, USP4, and HDAC2.

*TRPS1* is co-amplified with *MYC* in breast carcinomas with an increased proliferation rate [41], and silencing *TRPS1* reduces proliferation of BT474 cells [23]. Also, HDAC2 knockdown in breast cancer cells leads to inhibition of proliferation [42] and USP4 knockout results in the retarded growth of mouse embryonic fibroblasts (MEF) [20]. Our working model suggests that TRPS1 recruits USP4 to stabilize HDAC2 repressing the expression of *AES*, *Casp7*, *PERP*, and *ZW10* to confer tumor growth. Exogenous expression of *AES* suppresses the growth of LNCaP prostate cancer cells, while knockdown of *AES* promotes cell growth [43]. Inhibition or knockdown of *CASP7* impairs the growth of breast cancer cells [40]. Deficiency of *PERP* has been shown to promote tumor growth [44], whereas *ZW10* is essential in mitotic checkpoint control [45]. These scenarios fit well with our working model that TRPS1 recruits USP4 to stabilize HDAC2 and represses expression of *AES*, *Casp7*, *PERP*, and *ZW10* to confer tumor growth. It has been reported that TRPS1 represses the expression of RUNX2 [46] and ZEB2 [47]. Furthermore, reduced metastatic spread of triple negative breast cancer cells by TRPS1 has also been described [7]. How TRPS1 contributes to cancer metastasis needs to be further investigated. Nevertheless, our observations extend current knowledge of the importance of TRPS1 function in carcinogenesis by deciphering the TRPS1-UPS4-HDAC2 regulatory axis and uncovering how TRPS1 contributes to tumor growth.

Global loss of H4K16ac and H4K20me3 is a common marker of human cancer [48]. H4K16ac plays a critical role in the maintenance of active gene transcription, and its loss is important in the epigenetic silencing of some tumor suppressor genes in cancer [49]. H4K16ac has been shown to be a specific target of HDAC2 [16]. Our study has provided evidence that TRPS1, USP4, and HDAC2 are functionally connected through complex formation and that HDAC2 regulates gene transcription by deacetylating H4K16ac.

The ubiquitin system is critical in maintaining protein stability and level. So far, E3 ubiquitin ligase RLIM [50], Mcl-1 ubiquitin ligase E3 (MULE, also named ARF-BP-1) [51], and recently reported de-ubiquitinase USP4 [22] were documented to be involved in regulation of HDAC2 stability by the ubiquitin-proteasome system. However, we found that silencing of neither *RLIM29* nor *MULE* affected HDAC2 protein levels (data not shown). The specific mechanism of HDAC2 ubiquitination by ubiquitin ligases needs to be further investigated. Nevertheless, our observation that TRPS1 recruits USP4 to de-ubiquitinate HDAC2 extends the current knowledge on the regulation of HDAC2 stability by the ubiquitin system, contributing to tumor growth. An important regulatory step to counter the outcome of ubiquitination is by removing ubiquitin from ubiquitinated proteins by de-ubiquitinases [52]. Several studies have reported the importance of de-ubiquitination in stabilizing oncoproteins. For example, USP1 de-ubiquitinates and stabilizes two critical DNA repair proteins, FANCD2 and PCNA, and is involved in Fanconi leukemia [52, 53]; USP9x de-ubiquitates and stabilizes the pro-survival protein MCL1 [54]; USP37 is a de-ubiquitinase that regulates the cell cycle by de-ubiquitinating cyclin A [55] and c-MYC [56]. Thus, de-ubiquitinases are believed to represent alternative targets in the ubiquitin system for cancer therapies [57]. USP4, a ubiquitin-specific protease, was proposed to be a potential oncogene for decades [19]. Our observation that USP4 is recruited by TRPS1 to de-ubiquitinate HDAC2 and silence USP4, resulting in inhibition of tumor cell growth by TRPS1, is consistent with these notions elucidating the underlying molecular details of the oncogenic function of the TRPS1/HDAC2/USP4 axis in tumor growth.

## Conclusions

Our results suggest that the TRPS1-USP4-HDAC2 regulatory axis is implicated in carcinogenesis. HDAC2 implements the transcription repression program by deacetylating H4K16ac and contributes to tumor growth. Our data provide a mechanistic link between TRPS1, the ubiquitin system, and the histone modification system in cancer by revealing the TRPS1-USP4-HDAC2 regulatory axis that is involved in tumor growth. Furthermore, our results identified the novel non-transcription factor scaffolding function of the GATA family member TRPS1 in USP4-directed HDAC2 de-ubiquitination. Our findings suggest GATA transcription factors, ubiquitination regulators, and histone modifiers can serve as potential prognostic indicators and/or therapeutic targets of cancer.

## Additional files

Additional file 1: **Table S1.** RT-qPCR and ChIP-qPCR primer sequences. (DOCX 19 kb)

Additional file 2: **Table S2.** Sequences of siRNAs and shRNAs. (DOCX 16 kb)

Additional file 3: **Figure S1.** (**A**) MCF7, (**B**) T47D, and (**C**) BT474 exhibit insignificant alterations of HDAC1 protein level upon silencing of *TRPS1*. (**D**) MDA-MB-231 exhibits increased HDAC2 protein level upon overexpression of *TRPS1*. (**E**) MDA-MB-231 shows insignificant alterations in *HDAC2* mRNA level upon overexpression of *TRPS1*. (**F**) MDA-MB-231 shows increased HDAC2 protein stability upon overexpression of *TRPS1*. (JPG 2596 kb)

Additional file 4: **Figure S2.** (**A** and **B**) USP4 protein and mRNA levels were unaffected upon silencing of TRPS1 in T47D cell line. (JPG 1408 kb)

Additional file 5: **Figure S3.** (**A** and **B**) Silencing of *HDAC2* in T47D and MCF7 cells led to increased H4K16ac levels. (**C-E**) Silencing of *TRPS1* increased H4K16ac levels in BT474, T47D and MCF7. (**F**) USP4 could increase transcriptional repression activity of HDAC2. (JPG 3480 kb)

Additional file 6: **Table S3.** A, B Differential expressed genes upon silencing of *TRPS1* or *HDAC2* in MCF7 by RNA-sequencing. (XLSX 2609 kb)

## Abbreviations

ChIP: Chromatin immunoprecipitation
CHX: Cycloheximide
Co-IP: Co-immunoprecipitation
DMEM: Dulbecco’s modified Eagle’s medium
DMSO: Dimethylsulfoxide
HATs: Histone acetyltranferases
HDAC2: Histone deacetylase 2
KD: Knockdown
NURD: Nucleosome remodeling deacetylase
PBS: Phosphate-buffered saline
RT-PCR: Real-time polymerase chain reaction
siRNA: Small interfering RNA
TRPS1: Tricho-rhino-phalangeal syndrome 1
UDPD: Ubiquitin-dependent proteasomal degradation
USP4: Ubiquitin-specific protease 4
