# Supplementary material for: Tricho-rhino-phalangeal syndrome 1 protein functions as a scaffold required for ubiquitin-specific protease 4-directed histone deacetylase 2 de-ubiquitination and tumor growth
Source: Breast Cancer Res. 2018 Aug 2;20:83. doi: 10.1186/s13058-018-1018-7 (PMC6090974; doi:10.1186/s13058-018-1018-7)
Supplement: Supplementary file 1 — Table S1. RT-qPCR and ChIP-qPCR primer sequences. (DOCX 19 kb) [file 13058_2018_1018_MOESM1_ESM.docx]

**Additional file 1:Table S1** RT-qPCR and ChIP-qPCR primer sequences

| **Primers Sequence (5'-3')** | |
| --- | --- |
| **RT-qPCR primers:** | |
| TRPS1-RT-F  TRPS1-RT-R  HDAC2-RT-F  HDAC2-RT-R  Actin-RT-F  Actin-RT-R  CASP7-RT-F  CASP7-RT-R  ZW10-RT-F  ZW10-RT-R  AES-RT-F  AES-RT-R  PERP-RT-F  PERP-RT-R  IFT27-RT-F  IFT27-RT-R  TPM4-RT-F  TPM4-RT-R  SHISA2-RT-F  SHISA2-RT-R  **ChIP-qPCR primers:** | TCTACCAGAAGCTTCACTCG  CTCTCTAACGGGCTTCCATT  AAACTGCATATTAGTCCTTCAA  TGAGGTAACATGCGCAAATTTT  AACCCTAAGGCCAACCGTGA  GTCTCCGGAGTCCATCACAA  CGGTCCTCGTTTGTACCGTC  CGCCCATACCTGTCACTTTATCA  ATGGCCTCGTTCGTGACAG  CAGATAGCTTATCCACCTGGGTA  ACCCCAGCAACTCAAATTCAC  AAGCCGTAGGACATCTCGTAG  CTTCACCCTTCATGCCAACC  GCCAATCAGGATAATCGTGGCT  AGCTACACCCTGACAACAGGA  CCTTGCCAGCAGAGTCAAAAA  GAGGTAGCTCGTAAGCTGGTC  ACCGTTCTCTCTGCAAATTCAG  GGAGACCATCCCCATGATCC  AGCACAGAGAAATTCGTGGGC |
| CASP7-Qchip-F | TCCTCCCTTCCCTCCTAGCC |
| CASP7-Qchip-R | CAGGCAAGGGGTGGGGACCG |
| ZW10-Qchip-F | CCGCGTCGAAGCCGCCGGCC |
| ZW10-Qchip-R | GCGGCATTACCGCTGCCTTT |
| AES-Qchip-F | CCGCGTCGAAGCCGCCGGCC |
| AES-Qchip-R | GCGGCATTACCGCTGCCTTT |
| PERP-Qchip-F | TAGGACCCGCTGCCGCCGCC |
| PERP-Qchip-R | CCGCTCCGCTCGGCCCCGCG |
| IFT27-Qchip-F | TGCTCCCCGCCCAGCCCCTC |
| IFT27-Qchip-R | GAGGGGCTGGGCGGGGAGCA |
| TPM4-Qchip-F | AGGCGGTGAAACGCAAGATC |
| TPM4-Qchip-R | CGTACGTCGGAGGCCTTCTT |
| SHISA2-Qchip-F | AACTCTAAGCCACCCTTTGG |
| SHISA2-Qchip-R | TGCGCCCAGCGCGAAAGGGA |
| Untr4-Qchip-F | CTCCCTCCTGTGCTTCTCAG |
| Untr4-Qchip-R | AATGAACGTGTCTCCCAGAA |
