# Supplementary material for: Tricho-rhino-phalangeal syndrome 1 protein functions as a scaffold required for ubiquitin-specific protease 4-directed histone deacetylase 2 de-ubiquitination and tumor growth
Source: Breast Cancer Res. 2018 Aug 2;20:83. doi: 10.1186/s13058-018-1018-7 (PMC6090974; doi:10.1186/s13058-018-1018-7)
Supplement: Supplementary file 2 — Table S2. Sequences of siRNAs and shRNAs. (DOCX 16 kb) [file 13058_2018_1018_MOESM2_ESM.docx]

**Additional file 2: Table S2** Sequences of siRNAs and shRNAs

| SiRNA and shRNA Sequence (5'-3') | |
| --- | --- |
| Control siRNA  TRPS1siRNA#1  TRPS1siRNA#2  HDAC2 siRNA#1  HDAC2 siRNA#2  USP4 siRNA#1  USP4 siRNA#2  Control shRNA  TRPS1shRNA#1  TRPS1shRNA#2 | UUCUCCGAACGUGUCACGUTT  GUCCCUUGAAUGUAGUAAATT  GCACACAGCUGCUACAAAUTT  GCCUCAUAGAAUCCGCAUGTT  GGGUUGUUUCAAUCUAACATT  GCUGCUGGCCUUUCUUCUATT  GCAAAUGGUGAUAGCACUATT  CCGGTAAGGCTATGAAGAGATACCTCGAGGTATCTCTTCATAGCCTTATTTTTG  CCGGGCACACAGCTGCTACAAATGCCTCGAGGCATTTGTAGCAGCTGTGTGCTTTTTG  CCGGCGGACAAATATGACTTCACAACTCGAGTTGTGAAGTCATATTTGTCCGTTTTTG |
