# Supplementary figures and images for: Tricho-rhino-phalangeal syndrome 1 protein functions as a scaffold required for ubiquitin-specific protease 4-directed histone deacetylase 2 de-ubiquitination and tumor growth
Source: Breast Cancer Res. 2018 Aug 2;20:83. doi: 10.1186/s13058-018-1018-7 (PMC6090974; doi:10.1186/s13058-018-1018-7)

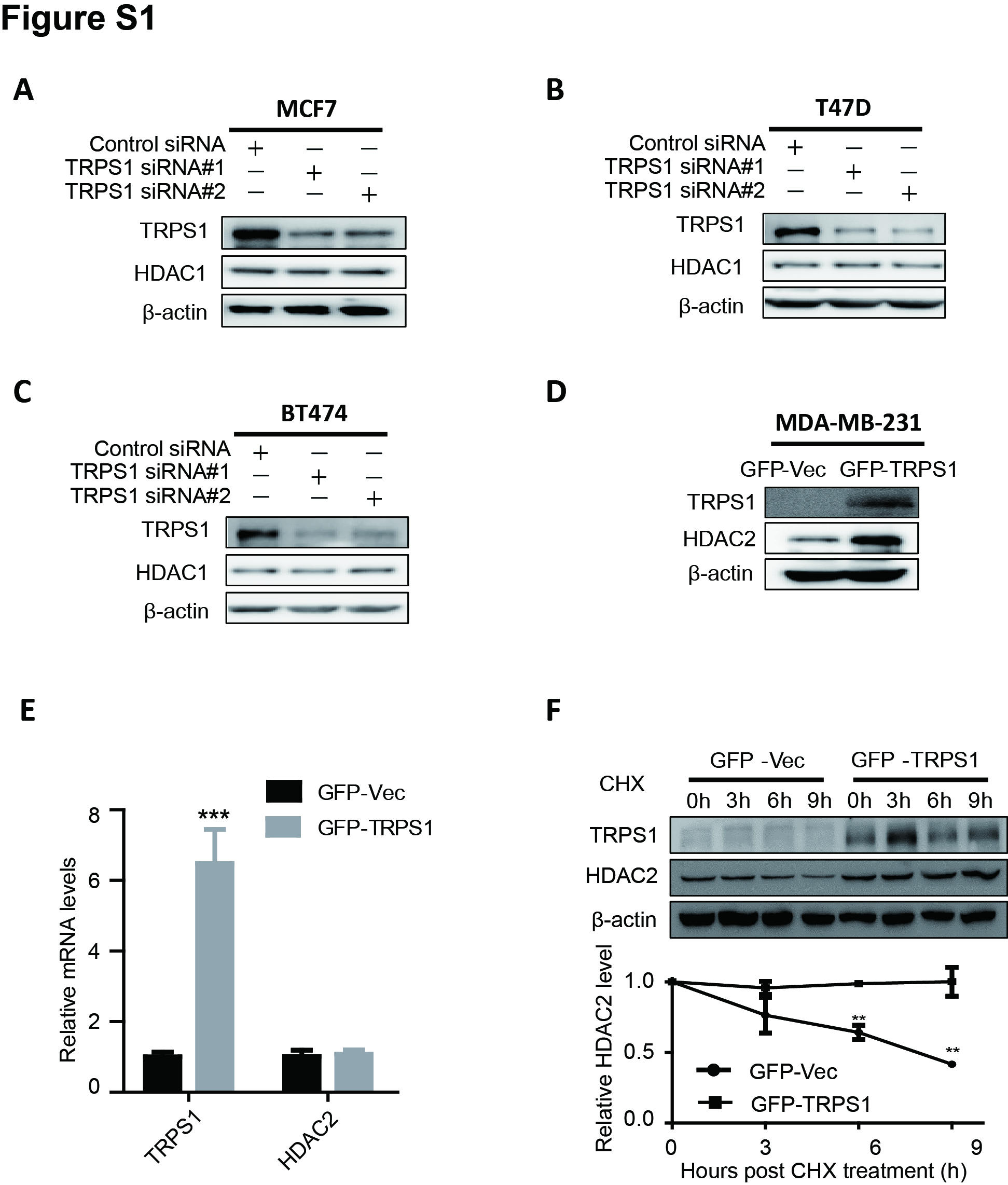

Supplement: Supplementary file 3 — Figure S1. (A) MCF7, (B) T47D, and (C) BT474 exhibit insignificant alterations of HDAC1 protein level upon silencing of TRPS1. (D) MDA-MB-231 exhibits increased HDAC2 protein level upon overexpression of TRPS1. (E) MDA-MB-231 shows insignificant alterations in HDAC2 mRNA level upon overexpression of TRPS1. (F) MDA-MB-231 shows increased HDAC2 protein stability upon overexpression of TRPS1. (JPG 2596 kb) [file 13058_2018_1018_MOESM3_ESM.jpg]

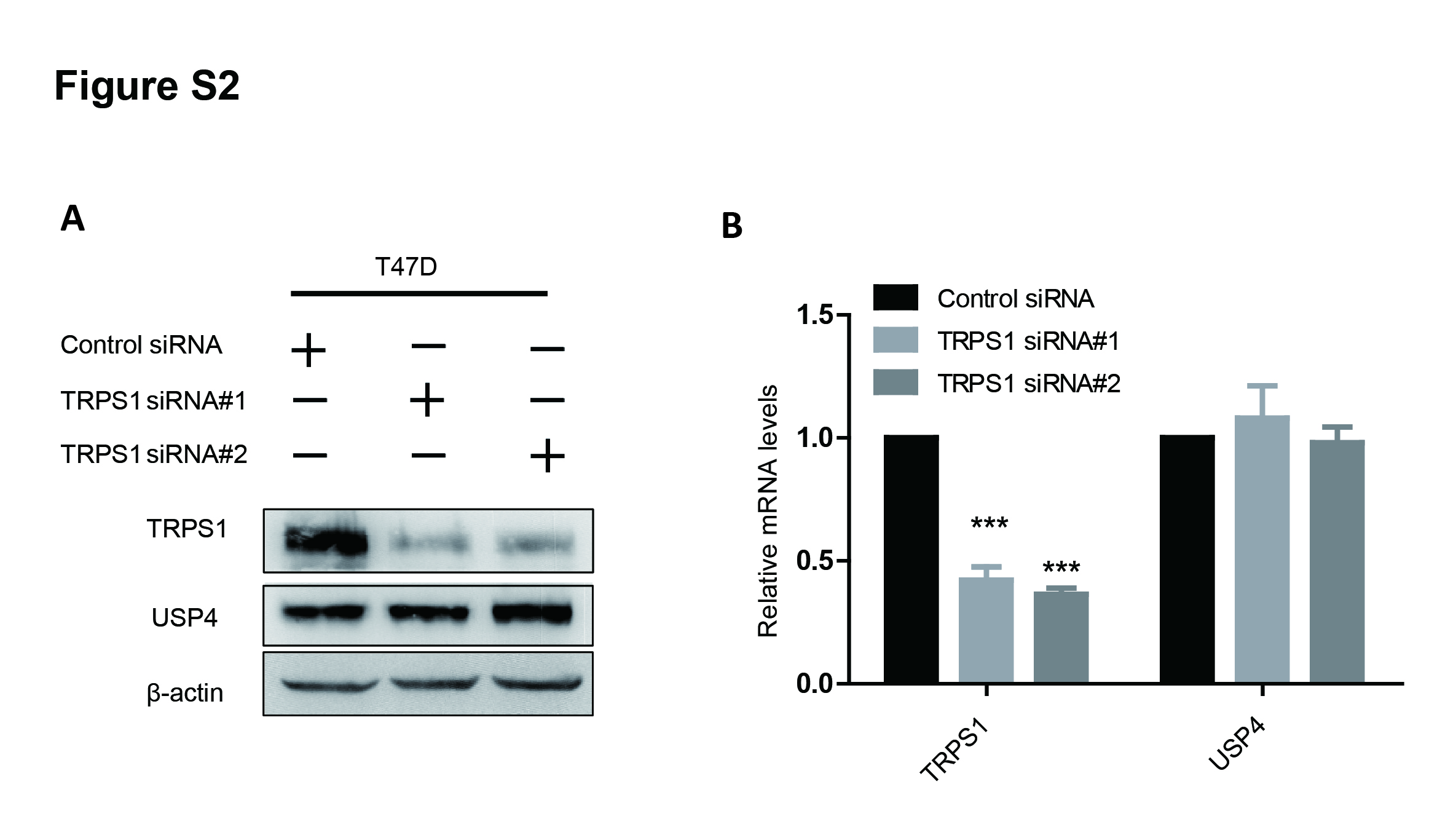

Supplement: Supplementary file 4 — Figure S2. (A and B) USP4 protein and mRNA levels were unaffected upon silencing of TRPS1 in T47D cell line. (JPG 1408 kb) [file 13058_2018_1018_MOESM4_ESM.jpg]

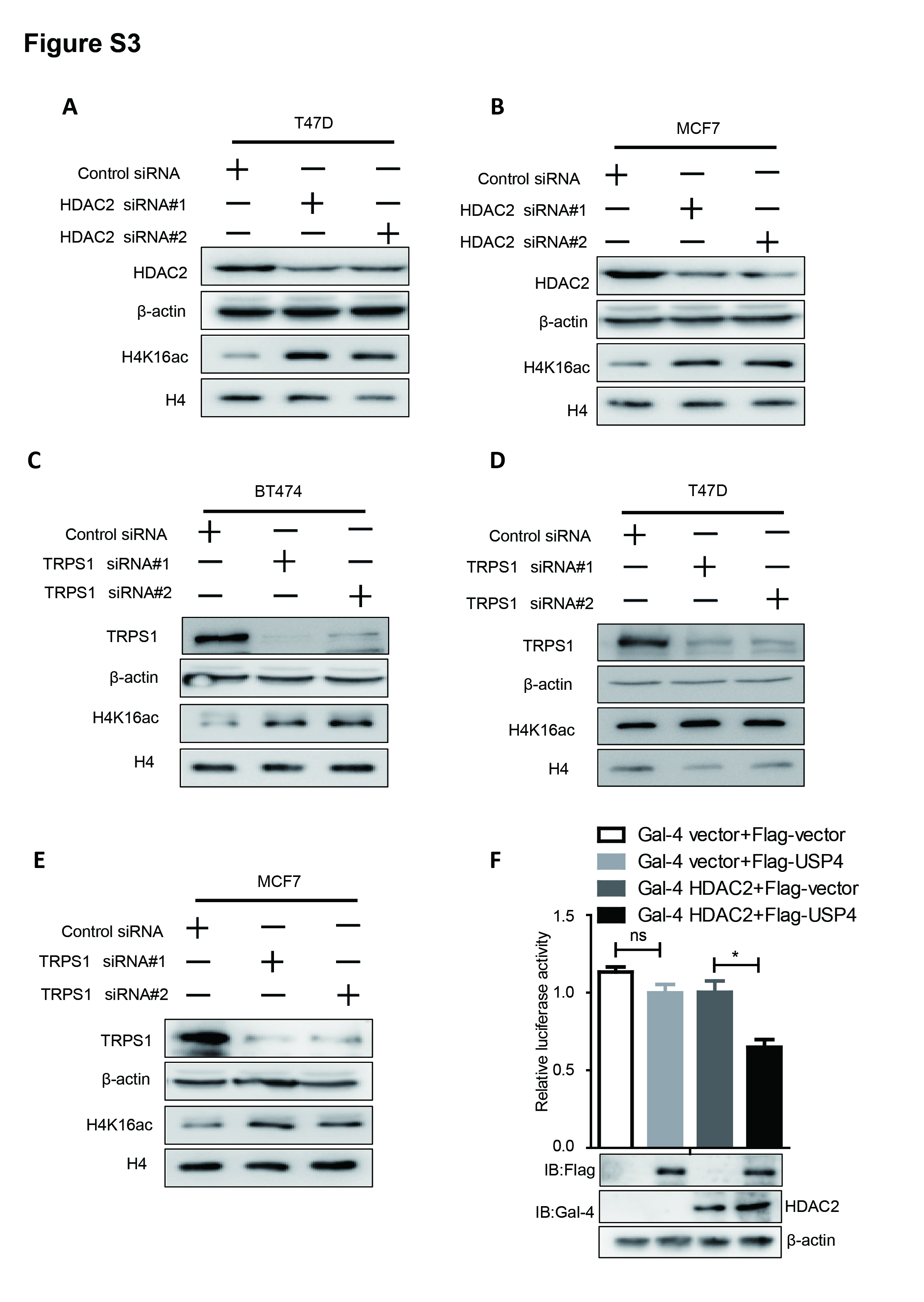

Supplement: Supplementary file 5 — Figure S3. (A and B) Silencing of HDAC2 in T47D and MCF7 cells led to increased H4K16ac levels. (C-E) Silencing of TRPS1 increased H4K16ac levels in BT474, T47D and MCF7. (F) USP4 could increase transcriptional repression activity of HDAC2. (JPG 3480 kb) [file 13058_2018_1018_MOESM5_ESM.jpg]
